# Host immune response against DENV and ZIKV infections

**DOI:** 10.3389/fcimb.2022.975222

**Published:** 2022-09-08

**Authors:** Shamala Devi Sekaran, Amni Adilah Ismail, Gaythri Thergarajan, Samudi Chandramathi, S. K. Hanan Rahman, Ravishankar Ram Mani, Felicita Fedelis Jusof, Yvonne A. L. Lim, Rishya Manikam

**Affiliations:** ^1^ Faculty of Medical & Health Sciences, UCSI University, Kuala Lumpur, Malaysia; ^2^ Department of Medical Microbiology, Faculty of Medicine, University of Malaya, Kuala Lumpur, Malaysia; ^3^ Department of Physiology, Faculty of Medicine, University of Malaya, Kuala Lumpur, Malaysia; ^4^ Department of Parasitology, Faculty of Medicine, University of Malaya, Kuala Lumpur, Malaysia; ^5^ Department of Trauma and Emergency Medicine, University Malaya Medical Centre, Kuala Lumpur, Malaysia

**Keywords:** immune response, Zika, Dengue, CD8+, CD4+, cytotoxicity, cross-reactivity

## Abstract

Dengue is a major public health concern, affecting almost 400 million people worldwide, with about 70% of the global burden of disease in Asia. Despite revised clinical classifications of dengue infections by the World Health Organization, the wide spectrum of the manifestations of dengue illness continues to pose challenges in diagnosis and patient management for clinicians. When the Zika epidemic spread through the American continent and then later to Africa and Asia in 2015, researchers compared the characteristics of the Zika infection to Dengue, considering both these viruses were transmitted primarily through the same vector, the *Aedes aegypti* female mosquitoes. An important difference to note, however, was that the Zika epidemic diffused in a shorter time span compared to the persisting feature of Dengue infections, which is endemic in many Asian countries. As the pathogenesis of viral illnesses is affected by host immune responses, various immune modulators have been proposed as biomarkers to predict the risk of the disease progression to a severe form, at a much earlier stage of the illness. However, the findings for most biomarkers are highly discrepant between studies. Meanwhile, the cross-reactivity of CD8+ and CD4+ T cells response to Dengue and Zika viruses provide important clues for further development of potential treatments. This review discusses similarities between Dengue and Zika infections, comparing their disease transmissions and vectors involved, and both the innate and adaptive immune responses in these infections. Consideration of the genetic identity of both the Dengue and Zika flaviviruses as well as the cross-reactivity of relevant T cells along with the actions of CD4+ cytotoxic cells in these infections are also presented. Finally, a summary of the immune biomarkers that have been reported for dengue and Zika viral infections are discussed which may be useful indicators for future anti-viral targets or predictors for disease severity. Together, this information appraises the current understanding of both Zika and Dengue infections, providing insights for future vaccine design approaches against both viruses.

## Introduction

Dengue is a disease transmitted by mosquitoes, with the primary vector being the *Aedes Aegypti* mosquito. Dengue virus (DENV) is an enveloped, single-stranded RNA virus belonging to the Flaviviridae family. The disease is endemic in at least 100 countries in the tropics and subtropics with 50 - 100 million infections and 22,000 deaths yearly, causing it to be the leading cause of illness and death in these regions (Waggoner et al., 2016). The number of DENV cases in the Americas, South-East Asia and Western Pacific has increased dramatically from 1.2 million in 2008 to 3.2 million in 2015 with many underreported and misclassified cases (Bhatt et al., 2013). Annually, around 390 million cases of DENV infections are encountered worldwide which results in 500,000 hospitalizations and 25,000 deaths, with children being the main casualty (Hasan et al., 2016; Khetarpal and Khanna, 2016). The disease which affects mostly tropical and sub-tropical countries, is spread through the bite of female *Aedes aegypti* or *Aedes albopictus mosquitoes.* Whilst the disease is prevalent in the Americas and Africa, almost 70% of the disease burden is in Asia, with highest incidence rates in India, China and Indonesia (Bhatt et al., 2013). In 2021, a total of 1,612 8509 cases were reported, with the majority of cases reported in Brazil (916 096), India (123 106), Vietnam (68 268), the Philippines (66 654) and Colombia (50 582). In the most recent report published on week 47 (ending 27 November 2021), 140 791 new cases have been reported, the majority from Brazil (52 446), Colombia (13 130), Pakistan (23 428), Peru (7 334), and Vietnam (6 964) (Prajapati et al., 2022). The classifications of DENV infection were revised in 2009 and patients were categorized as either having DENV without warning signs, with warning signs or severe DENV. A hallmark feature of severe DENV is hemorrhagic manifestations, where the patient may develop severe plasma leakage leading to shock, severe bleeding or severe organ impairment (World Health Organization, et al. 2009).

**Figure 1:**
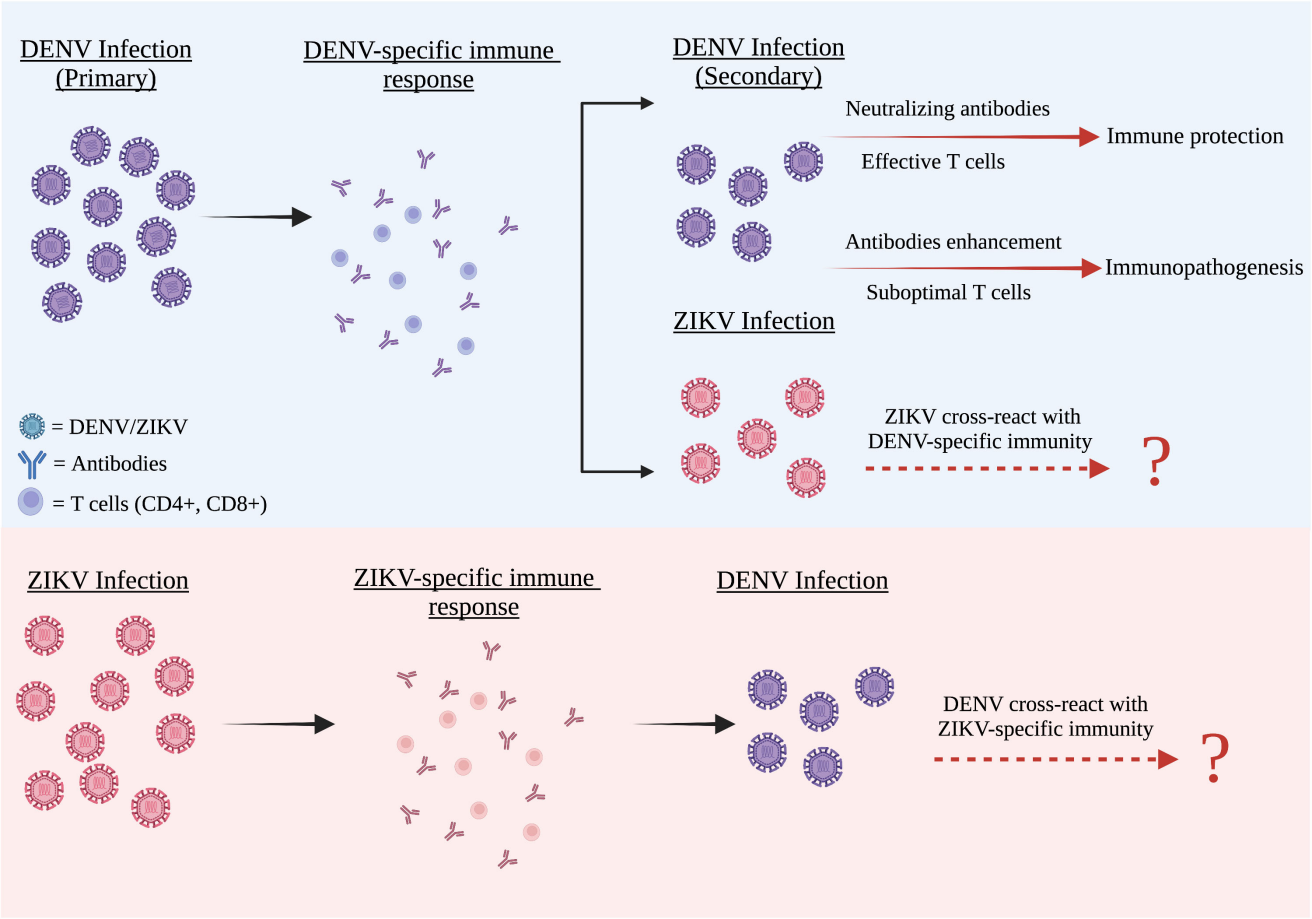
The cross reactivity between DENV and ZIKV infection. DENV and ZIKV were structurally and genetically highly similar to one another. Both these two flaviviruses have a strong humoral cross-reactivity with each other. After primary infection, all four DENV serotypes including ZIKV are partially cross-reactive with the DENV antibodies present in the DENV-infected patients. These antibodies often have lower avidities and weak neutralizing abilities. In both flaviviruses, weak antibody responses can promote an antibody-dependent enhancement (ADE) condition, whereas strong antibody responses neutralize the second virus infection and cause negligible disease.

DENV possesses an 11 kb genomic RNA which codes for three structural and seven non- structural proteins (Nedjadi et al., 2015; Alcalá et al., 2016). The three structural proteins are translated into the Capsid (C), Envelope (E) and Membrane (M) proteins, with the capsid forming a shell enclosing the viral RNA whilst proteins E and M are embedded within the spherical envelope which contains capsid and the virus as illustrated in **Figure 1**. Together these structural proteins protect and control the entry of the virus into host cells. On the other hand, the seven non-structural proteins are involved in viral replication, translation, and maturation of viral particles (Malavige et al., 2012). There are four closely-related DENV serotypes (DENV1, DENV2, DENV3 and DENV4) which cause DENV disease. Whilst being genetically similar and possessing some common antigens, these four viruses interact differently with host antibodies due to their structural differences (Thepparit and Smith, 2004). The existence of DENV serotypes adds to the complexity of the pathogenesis, diagnosis and development of vaccine of the disease. A previous DENV infection is identified as a risk factor for a more severe subsequent reinfection, especially when the two infections were caused by different DENV serotypes (Halstead et al., 2002). A phenomenon called the ‘antibody-dependent enhancement of infection’ is proposed to drive the increased severity observed in a DENV reinfection (Halstead, 2003). The enhancement of the infection is caused by the binding of the host antibodies that were generated from the previous DENV infection to the new DENV serotype from the reinfection. The antibody-DENV complex that forms, does not neutralize the virus. Rather, it facilitates a more efficacious invasion of the host immune cells by the virus. Consequentially, the viral load is amplified as host immune cells act as sites for viral replication. The amplification of viral replication triggers an intensified complement activation which results in cell death and tissue injury, which manifests as severe clinical manifestations often observed in a reinfection (Halstead, 2015). Despite the morbidity caused by DENV infection and its extensive disease burden, there are no vaccines for DENV currently. In addition, the responsibility of decision-making regarding the level of healthcare appropriate for individual patients lies entirely on clinicians despite the wide range of the infection spectrum (Ajlan et al., 2019). Often, the patients are treated on an outpatient basis or provided with supportive care depending on the signs the patients present with (Alcalá et al., 2016).

Similar to DENV, the Zika virus (ZIKV) is a *Flavivirus* transmitted by the same vector, the female *Aedes* mosquitoes. ZIKV infection was initially discovered in 1947 in rhesus monkeys in the Zika forest near Entebbe, Uganda (Simpson, 1964). However, by the year 1952, evidence of its infection in humans were reported in Uganda (Dye et al., 2016; World Health Organization, et al., 2016). Despite evidence of human ZIKV infection for more than 40 years, the first ZIKV outbreak occurred on the Yap Island Micronesia in 2007 (Duffy et al., 2009). They identified 49 confirmed and 59 probable cases of ZIKV disease with more than 73% of Yap residents 3 years of age or older were infected with ZIKV with *Aedes hensilli* being the predominant mosquito species identified. This represented the first outbreak of ZIKV outside of Africa and Asia. ZIKV then emerged in French Polynesia in 2013 with about 30,000 cases being recorded (Musso et al., 2014), followed by countries in South America such as in Chile and in Brazil where a rise in occurrences of microencephaly was noted (Tognarelli et al., 2016; Hennessey et al., 2016). This resulted in ZIKV infection being declared a global health emergency when the outbreak spread to 148 other countries (World Health Organization, et al. (2016); Alshammari et al., 2018). As with DENV, ZIKV also has a single-stranded positive-sense RNA genome, translated into a single polyprotein which is then cleaved into three structural proteins, capsid, pre-membrane (PrM), and envelope (E) and seven non-structural (NS) proteins: NS1, NS2a, NS2b, NS3, NS4a, NS4b, and NS5 (**Figure 1**). The virus is similar in structure to both DENV and West Nile Virus (WNV).

Both DENV and ZIKV infections shared similar symptoms including, fever, skin rashes, headache, malaise and joint pains (Sikka et al., 2016). The main difference here is that ZIKV infection lasts for a few days or weeks and then subsides, but in DENV infections it could go on for a few weeks and if untreated can lead to excessive bleeding. Severe DENV can sometimes be fatal. To note ZIKV infected individuals don’t get very sick and if they do are unlikely to die from the infection unlike DENV where fatality has been seen. However, ZIKV infection can be distinguished from DENV by the development of neurological disorders in ZIKV infections in the form of microcephaly or Guillain-Barѓe syndrome (GBS) (World Health Organization, 2015). Microcephaly is an *in-utero* brain development disorder that results in smaller heads in these infants whereas GBS is a form of muscle weakness caused by the immune system damaging the peripheral nervous system. The underlying mechanism involves an autoimmune disorder where the body mistakenly attacks the peripheral nerves and damages their myelin sheaths. This can be triggered by an infection. A recent study revealed a high level of peptide sharing between ZIKV polyprotein and human proteins related to myelin, demyelination, and axonal neuropathies (Koike, 2018). Both prenatal microcephaly and GBS syndromes have been associated with ZIKV infections as the number of incidences for the two conditions increased almost 20-fold during the ZIKV outbreak (CDC, 2014; Alhaeli et al., 2016; Basarab et al., 2016; Krauer et al., 2017). The other important difference between the two viruses is the mode of transmission, where ZIKV has been shown *via* case reports that transmission can also occur *via* sex, transplacental and blood transfusion (D’Ortenzio et al., 2016; Moreira et al., 2017; Zanluca et al., 2018). There are no well-accepted ZIKV- and DENV-specific vaccines for the prevention of the disease, to date. Thus, the treatment of these diseases relies mainly on supportive care, intake of fluid and administration of medicines for pain.

The first licensed DENV vaccine for the prevention of DENV, Dengvaxia, a live attenuated vaccine was approved and made available for use in 2015 (Villar et al., 2015) and was approved in Mexico, Philippines, and Brazil in December 2015, and in El Salvador, Costa Rica, Paraguay, Guatemala, Peru, Indonesia, Thailand and Singapore in 2016 (Capeding et al., 2014; World Health Organization, 2017; Prajapati et al., 2022). Dengvaxia is a chimeric yellow fever DENV vaccine without any DENV non-structural protein (Grifoni et al., 2017). The vaccine showed promising protection initially against DENV infection with vaccinated individuals having lower rates of hospitalization (Sabchareon et al., 2012; Villar et al., 2015; Halstead, 2018). However, long term studies on the vaccine efficacy showed increased incidences of severe DENV in vaccinated children (Modjarrad et al., 2017). In the analysis conducted by Sanofi (manufacturer) it was suggested that the vaccine may cause severe manifestations in those who have never been exposed to DENV but in others (who have pre-existing immunity) it was found to reduce risk of severe disease and hospitalization (Capeding et al., 2014). Another shortcoming of this vaccine is that it is effective against only a few DENV serotypes but not all. Phase 11 trials in Latin America and Asia showed that the vaccine efficacy was 64.7% and 56.5% respectively and this varied by serotype (Capeding et al., 2014). This clearly implicates that understanding the role of host immunity in these infections is important for designs of effective vaccines for the prevention of these two vector-borne diseases. With ZIKV, in 2017 a DNA vaccine consisting of a plasmid encoding the E and PrM was approved for Phase 2 clinical trials in humans (Modjarrad et al., 2017). Other types that have been developed include a purified inactivated vaccine, a live attenuated vaccine, an mRNA vaccine and a vector-based vaccine. At least 13 vaccine candidates under various platforms that have entered human phase I clinical trials with one entering phase II clinical trial. Some have been through preclinical studies and demonstrated significant promise for further development. Undoubtedly, this is considered a major success, although much work remains to be completed to bring a ZIKV vaccine to licensure and for public use. Several concerns need to be addressed before a successful ZIKV is deployed (Fernandez and Diamond, 2017; Modjarrad et al., 2017; Prajapati et al., 2022).

Both the innate and adaptive immune systems are involved in host responses against pathological infections such as DENV and ZIKV. The T lymphocytes, namely the CD4+ and CD8+ T cells form a key part of host immune responses in viral infections (Luckheeram et al., 2012). It has been shown that CD8+ T cells, also known as cytotoxic T cells, cause apoptosis of infected cells by having direct cytotoxic effects on virus-infected cells either through secretion of cytokines, production of cytotoxic granule or activation of the caspase cascade. The CD4+ (Helper) T cells support the activation and maturation of these CD8+ T cells. The CD4+ Helper T cells facilitate immune responses against infections by releasing cytokines which activate and augment antibody production by B lymphocytes, cytotoxic actions of T lymphocytes as well as amplify the responses of the cells of the innate immune system such as macrophages, neutrophils, basophils and eosinophils (Zhu and Paul, 2008). Although, conventionally, CD8+ is the major T cell mediating cytotoxic reactions against infected cells, recent research indicates that CD4+ T cells also exert similar cytotoxic effects as CD8+ T cells during DENV and ZIKV infection (Kennedy and Celis, 2008). This highlighted the importance of distinguishing the functions of the different subsets of T cells in the response and protection against DENV and ZIKV is key to prevention and treatment of these infections. This paper discusses the analogous and dissimilar immune responses of the human body against DENV and ZIKV infections. This includes discussion on certain immune correlates which could be used as future targets for antiviral agents or vaccine development.

## The innate immune response against DENV and ZIKV infectious diseases

Microbial entry into the body is restricted by physical barriers like skin and epithelial surfaces. These are considered as the first line of host defense. However, when these barriers are breached, the entry of the pathogens, into the host body activates the innate immune system which comprises of phagocytotic cells (such as dendritic cells, neutrophils, and macrophages) and the complement system (Warren, 2016). However, innate immunity does not result in any memory immune cells. The two primary outcomes of these immune responses are: 1) initiation of inflammation by phagocytes and macrophages and 2) antiviral defense by inflammatory cells and natural killer cells (NK). The antigen-presenting cells (APC) trigger the defense mechanism triggered through recognition of the antigen. This in turn, activates the effector immune cells which leads to a specific response against the target organism (Abbas et al., 2007).

Innate immune cells identify microbes by the specific lipids, carbohydrate and nucleic acid sequences present on the microbe which is necessary for its survival and virulence. There are unique molecular patterns that either present on the surface of the pathogen called pathogen-associated molecular pattern (PAMP) or on molecules released by pathogen-damaged cells called DAMP (damage-associated molecular pattern). These can be detected by immune cells which carry pattern recognition receptors (PRR). The interaction between PRR and PAMP/DAMP then activates a cascade of biochemical signaling important for the host immune response against the pathogen. Some of the most common PRRs for viral content detection are the toll-like receptors (TLRs), melanoma differentiation-associated gene 5 (MDA-5) and retinoic acid-inducible gene 1 (RIG-1). These PRRs detect and bind to the viral nucleic acid PAMPs present either on the membrane, in the lumen of intracellular vesicles or cytoplasm of infected cells (Kawai and Akira, 2009). More specifically, TLR3 identifies double-stranded RNA while TLR7 detects single-stranded RNA. This engagement between the TLRs and PAMPs prompts the production of cytokines (IFN-γ and IL-2) and chemokines (CCL 4, CCL5 and CXCL10) which exerts antiviral response by stimulating proteins and IFN regulatory factor (IRF 3) for the production of IFN-α and IFN-β (Costa et al., 2013; Uno and Ross, 2018). Inhibition of the virus infecting other cells occurs as type I IFN binds to their specific receptors (IFNARs) on the surface of infected cells (Pacsa et al., 2000). This activates a few pathways which blocks viral transcription, translation along viral RNA breakdown (Abbas et al., 2007; da Silva et al., 2019). These mechanisms act as defense against both DENV and ZIKV infection which is described in the studies of Silva et al. (2019) (da Silva et al., 2019) and Costa et al., (2013) (Costa et al., 2017). DENV fever patients have shown to produce high level of IL-2 along with IFN-γ (Brasier et al., 2012). Similarly, elevated levels of IFN-α, IFN-β, TLR 3, MDA-5, and RIG 1 expression are observed in ZIKV-infected patients (Nelson et al., 2020). The mosquito introduces DENV during a blood meal that is required for egg production as it injects the virus directly into the dermis with salivary fluid. However, some researchers opine that direct inoculation of the virus into the bloodstream may occur due to epidermal deposition (Martina et al., 2009). Other factors such as salivary components with reduced levels of pro-IL-1β and CXCL2 at the site of inoculation are suggested to reduce macrophage infiltration at the bite site (Hastings et al., 2019; King et al., 2020) and hence inducing innate immune cytokine responses here. DENV infectivity may also be amplified by CLIPA3, an *A. aegypti* salivary protein, which helps towards attachment of the virus to surface receptors on the cells, enabling cells to migrate by digesting the extracellular matrix (Conway et al., 2014). During this time several cells get infected which include Langerhans cells, dendritic cells, macrophages as well as keratinocytes and fibroblasts (Peña-García et al., 2017) *via* specific entry receptors. Some of the receptors implicated include C-type lectins, DC-SIGN, L-SIGN, CD300a, the mannose receptor, TIM-1, TAM, CD14, and glycosaminoglycans such as heparin sulfate (Ngono and Shresta, 2018). The structural domain III of DENV E protein is likely the receptor implicated on the virus (Cruz-Oliveira et al., 2015). Inside these cells, upon recognition of PAMPs (mainly ssRNA and dsRNA) by the host’s PRRs such as MDA5, RIG-I, TLR3, and TLR7, an antiviral response is triggered. This is characterized by type I interferons and transcription factors, IRF3, IRF7, and NF-κB (Uno and Ross, 2018; Tremblay et al., 2019). This then directs the transcription and secretion of IFN-α and IFN-β. Acting in an autocrine and paracrine manner, more cells get infected. The production of TNF initiates the synthesis of the IL-1β pyrogen, responsible for appetite suppression myalgia and pyrexia (Schaeffer et al., 2015). Clinical symptoms observed such as fever, chills, headache, and fatigue are probably due to Type 1 interferon (Todd and Goa, 1992; Sleijfer et al., 2005; Owen et al., 2013; Torkildsen et al., 2016). In ZIKV, several studies carried out (Hamel et al., 2015; Bayer et al., 2016; Quicke et al., 2016; Chaudhary et al., 2017) using human cells assessed IFN responses and showed that ZIKV infection resulted in the generation of type I (α, β), type II (γ), and type III (λ) IFN accompanied by the activation of several IFN-stimulated genes (ISGs). It was shown that STAT2, a signaling molecule involved in the type I IFN pathway, is degraded by ZIKV NS5, in human dendritic cells (Bowen et al., 2017) but not in mice which limits immune signaling and enhances viral replication. The study of ZIKV infection in human dendritic cells indicated that there is antagonism of STAT1 and STAT2 phosphorylation suggesting other mechanisms maybe involved.

Several studies implicate a subversion of the interferon response as apart from the skin the virus is also found in several types of cells beyond the site of infection. Several DENV non-structural (NS) proteins impede activation and downstream signaling of interferon. It is said that NS2a, NS4a, and NS4b block phosphorylation of TBK1 and this prevents transcription of IFN-β (Dalrymple et al., 2015). NS2b/3 protease complex on the other hand, targets Sting, an intracellular DNA, suggesting that STING plays a key role in the inhibition of DENV infection which is said to circumvent the induction of interferon (Kao et al., 2018). Other mechanisms by which interferon induction is circumvented include methylation of the 5’ cap of the DENV genome by NS5 2’-O-, which prevents detection by RIG-I (Kao et al., 2018), as well as NS4a inhibiting the binding of RIG-I to DENV (He et al., 2016). This occurs *via* binding of NS4a to the CARD-like and transmembrane domains of MAVS.

Whilst most of the antagonism towards IFN response in DENV infection occurs upstream of by inhibiting IFN synthesis, some inhibition targets the generated IFNs directly, as cells such as plasma dendritic cells can produce IFN without being infected themselves. There are 7 non-structural proteins in DENV and of these, five proteins function to target the interferons. This focus by the virus NS regions indicates how important interferons are for limiting DENV infections. The above mechanisms utilized by the virus enables replication, producing virions that infect cells beyond the point of entry.

ZIKV as we know, is cleared from the blood but however persists in saliva, breast milk, semen, urine, and the central nervous system (CNS) for months. Studies have also showed that ZIKV replicates in many different cell types (human endothelial, epithelial cells PBMCs, astrocytes, microglial cells). This broad cell tropism is also seen in *in vitro* cell lines (Jabrane-Ferrat and Veas, 2020). The host recognizes the virus hence the fast clearance in blood by induction of IFN cascade in an autocrine and paracrine manner as it occurs for dengue too with subsequent activation of various transcription factors (JAK/STAT (STAT1 and STAT2). However, ZIKV has multiple evasion strategies. Even though recognized by PRRs (MDA5; RIG-1) (Tappe et al., 2016; Hou et al., 2017), the virus carries several components such as the structural E protein, NS proteins, genomic and non-coding viral RNA, that can counter IFN antiviral functions by modulation of the interferon pathway and complement antagonism (Conde et al., 2017). The E protein, although being a strong inducer of the humoral immune response, is said to not only inhibit IFN production but the antibodies triggered have poor neutralizing capacity. Also, the target receptor for ZIKV, Axl, once activated suppresses type I IFN signaling. The non-structural proteins also are able to modulate antiviral IFN signals. A natural, evolutionary NS1 mutation in the Asian ZIKV lineage has been described to inhibit IFN-β production in human cells. Here similarly NS1 and NS4b target type I IFN production while NS2a downregulates the promoter activity of IFN-β and suppresses RIG-I and MDA5 (Kumar et al., 2016; Xia et al., 2018). In ZIKV infection, NS3 blocks RIG-I- and MDA5-mediated IFN antiviral actions. These multiple evasion mechanisms of viral sensing results in a favorable environment for viral replication. Other non-structural proteins that are implicated include the highly conserved NS5 which apart from affecting interferon production also impairs sensing of genomic RNA and causes proteasomal degradation STAT2, an IFN-regulated transcriptional activator. This reaction allows ZIKV to evade the IFN-mediated innate immunity. A summary of host innate immunity against DENV and ZIKV is given in **Table 1** below.

**Table 1:** Host innate immunity against DENV and ZIKV.

Virus	Virus factor	IFN Pathway	Host target	References
DENV	NS2A and NS4B	RLR, TLR, cGAS-STING	Reduction of IRF3 protein production and inhibition of TBKI	(Dalrymple et al., 2015)
ZIKV	NS5	RLR, TLR, cGAS-STING	IRF3 binding	(Kumar et al., 2016; Xia et al., 2018)

## Cells of the innate immune system

Mast cells have also been implicated in DENV infection. These cells release CXCL2 which promotes recruitment and activation of neutrophils from the periphery thus contributing to the clinical rash often seen during infection with DENV (Zhang et al., 1992; Abraham et al., 1997; Nakamura et al., 2009; Mishra et al., 2018). King et al. (2000) (King et al., 2000) has shown that mast cells have permissive actions to DENV infection *via* ADE while Brown et al. (2011) (Brown et al., 2012) showed that this resulted in a robust IFN expression. This suggests that Type 1 IFNs and chemotactic factors released upon degranulation induce an acute immune activation which further recruits local tissue macrophages, circulating monocytes, neutrophils and NK cells to the site (St. John, 2013). Human studies of subjects with rashes demonstrate degranulation of mediators such as chymase (Tissera et al., 2017), histamine (Tuchinda et al., 1977), VEGF (Furuta et al., 2012), and tryptase (Rathore et al., 2019). This implicates the important role played by the virus in inducing release of mast cell mediators that reflects on the clinical symptoms of DENV disease observed. Many studies have been conducted with drugs targeting vasoactive mast cell mediators and suggest that this may be one mode of treatment in DENV disease (Tan et al., 2019). It must also be emphasized that myeloid cells are also recruited to the site of inflammation *via* CCL2, CCL20 and IL-1β (Duangkhae et al., 2018). On the other hand, CCL1 and CCL5 facilitates extravasation (Shi and Pamer, 2011) and CCL2 facilitates recruitment of plasma dendritic cells to inflamed skin (Swiecki and Colonna, 2011). Tropism may differ depending on the cell type and receptors they bear (Dejnirattisai et al., 2011). Other cells such as neutrophils, which upon activation by DAMPs, PAMPs and pro-inflammatory cytokines, as well as complement split products like C5a, C3a, CXCL2 and TNF (Silvestre-Roig et al., 2016) result in the release of NETs which in excess is said to contribute to pathogenesis of several different viral infections. However, more thorough research is needed.

Meanwhile, another component of innate immunity, the natural killer cells help eliminate infected cells. Virus-infected cells lose their ability to synthesize MHC- class I which is then detected by the NK cells (Warren, 2016). In addition, NK cells are also activated by IL-12 and TNF-α cytokines, secreted by macrophages with engulfed virus or infected cells. Once activated, the NK cells trigger apoptosis in the target cells by secreting perforins and granzymes which degrade cell membrane proteins, or through Fas-FasL interaction (Warren, 2016). These mechanisms of virus elimination by NK cells are a common defense approach also observed in the innate immune response against DENV and ZIKV (Ngono and Shresta, 2018). Recently CD69+CLA+ CXCR3+ CCR5+CD56 +NK cell recruitment to the skin during acute DENV infection was shown in human skin studies of DENV infected patients (Homchampa et al., 1988; Chen et al., 1999; Laoprasopwattana et al., 2007; Zimmer et al., 2019). Studies with Brazilian patients also indicate increase in circulating NK cells with early activation markers and increased expression of cytotoxic granule, TIA-1 with significant elevation of IL-15, which activates NK cells (Azeredo et al., 2006).

As we also know DENV migrates towards the draining lymph nodes upon stimulation by IL-1β and TNF (Schmid and Harris, 2014; Rathore et al., 2019). It has been established that there is an intrinsic incubation period of approximately 5-6 days prior to viraemia (Chan and Johansson, 2012). The innate arm initiates the adaptive immune response causing further viral replication in recruited and lymph node-resident mononuclear phagocytes which then allows infectious DENV virions to enter the peripheral blood (Castillo et al., 2019). However, in general it is agreed that the critical players are the monocytes which are the main target (Halstead and O'rourke, 1977; Durbin et al., 2008; Zanini et al., 2018). This is seen at a higher frequency in secondary DENV infection as a result of ADE. This model also shows that TNF secretion increases (Halstead and O'rourke, 1977) which then drives severe disease. Elevation of liver enzymes ALT and AST observed is a result of increased dissemination to Kupffer cells of the liver and macrophages of the spleen which release more TNF (Balsitis et al., 2009; Fernando et al., 2016).

In addition to these innate responses, complement system also plays a role in fighting against viral infection by binding to infected cell surface proteins *via* the mannose-binding lectin associated with mannose glycans. Such interaction activates the classic complement cascade which initiates lysis of the infected cell along with the recruitment of phagocytes for further action (Uno and Ross, 2018). This antagonistic role may either limit viral replication and protect the host or result in an exaggerated inflammatory response which increases disease severity by its disproportionate activation. Clinical studies have shown excessive consumption of complement proteins and increased levels of complement activation products enhances vascular permeability, thereby influencing disease severity. Therefore, the outcome of flavivirus infections is evidently determined by the complement system. Downregulation of complement proteins by viruses have consequences and research in this area will provide insights into regulatory immune responses which would contribute to improving strategies for design of anti-viral inhibitors and vaccines. Although, innate action is effective, viral antigen can still evade the immune cells and continue to infect healthy cells. This is where the adaptive immunity is not only essential but necessary to control and eliminate the microbe efficiently.

In general the interferon system is the central mediator of protection against DENV and ZIKV, implicating roles for TLR3 and TLR7 in DENV infection in cells (Wang et al., 2006; Nasirudeen et al., 2011), IRF-5 (Lazear et al., 2013), IRF-1 (Brien et al., 2011) in ZIKV and, MyD88/TRIF and IRF-5/IRF-1 in the signaling events of both viruses. However, the past decade of research has indicated that DENV antagonizes the signal transduction pathways of type I interferon system (Suthar et al., 2013), by manipulation of the cytosolic DNA receptor cGAS and its adapter STING (Aguirre et al., 2012). This said to occur with DENV NS2A, NS4A, and NS4B which suppress STAT1 activation and downstream ISG expression (Munoz-Jordan et al., 2003). Studies to date indicate that DENV NS5 may be the most potent antagonist of type I interferon signaling. Despite this the precise mechanisms by which the type I interferon system mediates antiviral defense are as yet to be fully understood. Both DENV and ZIKV likely have similarities in the many aspects of their interactions with the type I interferon system. There are however differences in the biology of DENV and ZIKV and hence differences in this aspect will vary between the viruses. This may likely be due to the different tropisms of the 2 viruses. Importantly, identifying other components of the innate immune system, for example the complement system, γδ T cells, cytokines released by other cells like mast cells, NK cells and the vasoactive mediators and various types of innate lymphoid cells, is essential for understanding the precise pathogenic role.

## Adaptive immune responses

During any infection, antigen presenting cells (APC) (dendritic cell and macrophages) work as the initiator of the immune response. APC cells patrol around the body in search of pathogen. These cells engulf any encountered microbe and digestion into peptide remains followed by presenting those peptides on their surface (Tsang et al., 2003). The surface peptides later bind to MHC molecules to form MHC class II complex (Luckheeram et al., 2012). This complex is very important for immunity against pathogens. Naïve CD4+ T lymphocytes identify such complexes and interacts with them causing CD4+T cells to activate, proliferate and differentiate into active effector cells with the support of cytokines (Tubo and Jenkins, 2014).

Differentiated effector CD4+T cell eliminate the microbes and infected cells and later die while some remain active as memory cells for long term protection in blood. These memory cells are located around the body based on their functions (Tubo and Jenkins, 2014). The 2 main memory cells: central memory T cells and effector memory T cells migrate to the site of secondary infection by expressing surface receptor CD62L and CCR7 (Gasper et al., 2014). The activated T cells are differentiated into different subsets each with their specific functions during infection based on lymphokine and cytokine production (Sehrawat and Rouse, 2017). Differentiation of CD4+ T cells occurs by the transcription stimulation of transcription factor STAT5 which is activated by IL-2 mainly for Th1 and Th2 differentiation (Tripathi and Lahesmaa, 2014) categorized the subsets into two dichromatic groups of Th1 and Th2 which was later increased into more divisions of Th9, Th17, Treg (regulatory helper) or T_fh_ (follicular helper) according to Coghill et al. (2011) (Coghill et al., 2011).

### Adaptive immune response during DENV

Our past experiences with this virus suggest that both can be protective or disease enhancing with subsequent exposures to different DENV serotypes. Those with asymptomatic infection appear to have a higher percentage of activated T cells as compared to those with symptomatic infection. Cross-reactive antibodies (Abs) produced by plasma blasts bind to conserved epitopes of the DENV family as seen during a secondary infection. These cross-reactive Abs may exacerbate disease severity when present at sub-neutralizing concentrations, however, at higher concentrations, they appear to render increased protection. CD4+T cells that bear the phenotype CX3CR1, CD57, perforin, granzyme and TBET with a cytotoxic effector phenotype are found at higher levels in individuals with more than one previous DENV infection (Weiskopf and Sette, 2014). Of the T cell epitopes, IgG+ memory B cells correlated negatively with PD-1+ DENV-specific CD8+T cells. B cells with higher levels of PD-L1 have been described to limit differentiation and function of T follicular helper cells (T_fh_s), but possible interactions between T_fh_s and PD-1+ CD8+T cells is not known. It was reported that PD-1+ CD8+T cells are defective and incompetent at clearing viral infection and have been noted to be present in early DENV infection (Rouers et al., 2021). Thus, may have a negative impact on memory establishment.

CD 8+ T cells are responsible for the cytotoxic effect caused by interaction through Fas/FasL or by the release of cytotoxic granules identified as granzymes and perforins. These cytotoxic granules enter infected target cells resulting in arrest in viral protein production which leads to cell apoptosis (Martina et al., 2009). Meanwhile, many hypotheses indicate that protection of the body against DENV also includes components of the complement system (Martina et al., 2009) but researchers have found that the Antibody dependent enhancement (ADE) mechanism appears to be the most rational (Halstead, 2015). During DENV infections, fever shifts from mild to severe DENV hemorrhagic fever which shifts T cell response from Th1 to Th2 (Chaturvedi et al., 2000). At the initial stage of infection, the fever caused is considered as mild which becomes severe with secondary infection by DENV which is said to be due to the presence of non-neutralizing antibodies which enhances entry into cells. The “original antigenic sin” theory states the presence of weak cross-reactive T cells which recognizes the DENV virus and get activated for proliferation (Rothman, 2011). The activated T cells stimulates secretion of proinflammatory cytokines such as IFN-β, TNF-α, which then leads to plasma leakage which is the hallmark of severe DENV (Friberg et al., 2011). Furthermore, high levels of IFN-y/IL-4 in severe DENV patients indicates a strong link between ADE mechanism as Fcγ receptors accept more non-specific Ag-Ab complex on antigen presenting cells, thus increasing cross reactive responses (Singla et al., 2016), resulting in IFN-γ secretion (Kuczera et al., 2018). Meanwhile, other T cell subsets also act against DENV infection such as Tregs which increases and expands in acute DENV infection (Jayaratne et al., 2018). Although, levels of Tregs increase, the role in DENV infection needs further understanding and analysis as at high levels, immune activation and cytokine production is suppressed (Luhn et al., 2007). Studies also report that Treg cells secrete effector molecules which result in cytotoxic activity with aid of perforin and granzyme (Tian et al., 2016). What is also seen is an increase in the levels of many cytokines in the blood such as IL-6 (Rachman and Rinaldi, 2006), IL-18, IL-4 (Mabalirajan et al., 2005) and IL-10 (Pandey and Pandey, 2015) along with apoptosis markers (Zain et al., 2017). Soe et al. (2018) (Soe et al., 2018) stated that platelets show no relation with DENV infection in DENV patients. Thus, these markers can be used to determine the severity of DENV fever.

On the other hand, other immune cells such as dendritic cells and monocytes are known to be targets of DENV. Cross- reactive T cells cause these infected cells to secrete more IFN-γ (Libraty et al., 2001). Natural killer cells have also been shown to increase in patients with DENV fever indicating FAS-mediated apoptosis and cytotoxicity occurs due to activation of CD69 and TIA-1 cytotoxic markers (Gandini et al., 2017). All these immune cells and cytokines are observed to be more in skin blisters of patients than in blood as DENV infection is initiated at the site of the mosquito bite in the host skin (Halstead, 2017). Studies have also shown that T cells are attracted towards those sites that filter lymph fluid (Duyen et al., 2017). According to Immune Epitope Database query for DENV (ID: 12637), 2191 epitopes are present in the virus surface (Weiskopf and Sette, 2014) with 825 restricted HLA class I (Angelo et al., 2017) and 1345 HLA class II NS3 protein (Grifoni et al., 2017) but the most dominant one is NS3 protein, NS5 protein (Weiskopf and Sette, 2014), HLA class I and HLA class II (C_50-64_ and C_72-86_) (Weiskopf et al., 2013). NS3 and NS5 are the largest encoded proteins in the DENV viral genome (Duangchinda et al., 2010; Mathew et al., 2014).

Apart from the cytotoxic effects exerted by CD8+T lymphocytes, CD4+T lymphocytes have been shown to be cytotoxic by Billing *et al.* (1977) (Billings et al., 1977) and Dennert *et al.* (1981) (Dennert et al., 1981). T cell subsets release the cytokine IL-2 that enables the differentiation of CD4+T cells into CTL cells. IL-2 initiates expression of Eomesodermin along with RunX3 transcription factors which causes the development of cytotoxic T cells (Marshall and Swain, 2011). Co-stimulatory signals also help towards CTL cell differentiation (Qui et al., 2011). Another theory of CTL cell generation was stated in the study by Takeuchi & Saito (2011) (Takeuchi and Saito, 2017), where they have implicated a molecule, which restricted T cell release of IFN-γ which is needed to increase the level of granzymes in CD4+CTL differentiation (Juno et al., 2017). This molecule, CRTAM, could also be used as a marker as it recognizes NS1 and NS3 proteins on the surface of DENV infected cells during the acute phase of DENV infection (Kurane et al., 1991). These proteins are produced by cleaving the viral polypeptide during the transcription process of the viral genome (Duangchinda et al., 2010). The target cells are said to be eliminated by CTL CD4 cells *via* two mechanisms according to different researchers (Appay et al., 2002), *via* perforin and granzyme cytotoxic mechanism (Hildemann et al., 2013) or *via in vitro* experiments conducted on DENV infected mice showing a positive CTL CD4 cytotoxic effect (Rothman et al., 1996; Yauch et al., 2010). The latter mechanism involves FasL pathway where both pathways contribute to the destruction of target and bystander DENV infected cells (Gagnon et al., 1999; Malyshkina et al., 2017). After the elimination of DENV infected target cells, memory CTL CD4 cells express DENV specific HLA class II proteins for protection against secondary infection leading to severe DENV disease (Weiskopf and Sette, 2014; Juno et al., 2017).

### Adaptive immune response during ZIKV

In the studies of Lucas et al. (2018) (Lucas et al., 2018), Scott et al. (2018) (Scott et al., 2018) and Winkler et al. (2017) (Winkler et al., 2017), mouse models were used to investigate the response of CD8+ and CD4+ T cells to ZIKV infection. T cell responses were observed on day 7 post onset of symptoms (POS) which peaked on day 21 with memory cells appearing on day 148 POS (Ricciardi et al., 2017). Among the memory cells, CD8+ T cells were detected at higher levels than CD4+ memory T cells. The T cells detected during the infection phase were highly polyfunctional and were stimulated with pools of 11 overlapping peptides from all ZIKV proteins (El Sahly et al., 2019). Apart from cells, T cell cytokines (IFN-y and IL-12) and chemokines (CCL5) were also detected (Cimini et al., 2017; Lum et al., 2018; Jurado et al., 2018). The CD4+T cell cytokines were produced upon stimulation with NS1, NS3 and NS5 protein peptides of capsid and envelope protein while CD8+T cells responded to NS3, NS4B and NS5 proteins (El Sahly et al., 2019). NS5 protein is common for both DENV and ZIKV detection as it is a highly conserved region for all Flaviviruses (Ngono and Shresta, 2018). Studies of Elong et al. (2017) (Ngono and Shresta, 2018) and Pardy et al. (2017) (Pardy et al., 2017) using mice models infected with ZIKV, confirmed the role of cytotoxic Th_1_ CD4+T cells being the major cell type detected during the ZIKV response (Ngono and Shresta, 2018). Specific CD4+T cells polarize to Th1 phenotype while specific CD8+T cells get activated to an effector phenotype at the peak of the adaptive response. This causes production of effector cytokines and cytolytic molecules, such as IFN-γ, tumor necrosis factor (TNF)-α and IL-2 and T-bet, without inducing substantial Th2 or Th17 responses. A novel ZIKV CD8+T cell epitope in the envelope protein which is conserved across 99% (Balsitis et al., 2009; Zanini et al., 2018) of all full-length ZIKV polyprotein sequences is available on Genebank, making it a powerful epitope for vaccines against ZIKV. Although, findings have confirmed this statement, it still requires further investigation on the cytotoxic effect and mechanism of CD4+CTL cells in ZIKV clearance in the body.

With regard to the antibody response, it is imperative to note that ZIKV infections also occur in many areas where other flaviviruses cocirculate. The E protein is the major target of antibodies as it induces long term immunity which in subsequent infections result in cross reactivity among flaviviruses which have been found to result in disease susceptibility and severity. Using printed microarrays (Giraldo-García and Castaño-Osorio, 2019) showed that in areas where flaviviruses are endemic, the antibodies recognized E proteins from many flaviviruses, in a heterotypical manner rather than in a homotypical manner especially DENV, indicating a strong influence of infection history on immune responses. Hence this limits the usage of current serological assays and also complicates vaccination strategies. Newer techniques and detection methods are direly in need to guide definitive and accurate diagnosis of these infections. A summary of host adaptive immunity against DENV and ZIKV is given in **Table 2** below.

**Table 2:** Host adaptive immunity against DENV and ZIKV.

Virus	T Cells	Immune Markers	Function	References
DENV	CD8+ T cells	CLA	Recruiting T cells to skin area providing instant protection	(Azeredo et al., 2006)
	CD8+ T cells	CD69, HLA-DP, DQ, DR, CD38, cytotoxic granule TIA-1, VLA-4, ICAM-1, LFA-1CD44, CD11a	Activate T cells to eliminate virus by inducing inflammation	(Azeredo et al., 2006)
	CD8+Tem (CD45RA-CCR7-) and Temra (CD45RA+CCR7-)	IFN-γ, CCL3/CCL4, CD69, CRTAM, TNF-α, CTLA-4, ICOS, LIGHT, IRF4, IRF8, SLAMF7, KIR2DL3	Activate T cell, proliferation and polyfunctional properties	(Chaturvedi et al., 2000)
	CD4+Temra CD45RA+CCR7-	CX3CR1, serine protease granzyme, IFN-γ	cytotoxic, protective role in DENV with HLA DR allele	(Munoz-Jordan et al., 2003; Zain et al., 2017)
	CD4+CD25+FoxP3+ Treg	CTLA-4	Production of immune-suppressive cytokines	(Martina et al., 2009; Rouers et al., 2021)
ZIKV	CD8+1FN-y+ T cells	IFN-γ, TNF-α, Granzyme B	Initiate Th (Th1, Th2, Th17 and Th9) responses	(Tappe et al., 2016; Grifoni et al., 2017)

Half a century ago studies in Thailand have indicated sufficient evidence that the DENV is capable of modulating host immunity to the extent of causing severe disease. This is due to the cross-reactivity among flaviruses and of multiple infections with different serotypes and caused by memory of previous infections. Sufficient neutralization is key here and in instances of low levels of such antibodies ADE will occur. As stated by many researchers a strong antibody response will neutralize the second virus and result in negligible disease, while minimal levels can generate ADE conditions. As for T cells, a strong response is required to abrogate the effects of ADE. If the T cell response is attenuated by host factors such as HLA or inadequate antigen presentation, then ADE will progress. With this backdrop any vaccine developed needs to address all branches in a collective manner

### Cross reactivity between DENV and ZIKV infection

Both DENV and ZIKV viruses share a close antigenic relationship (Harrison, 2016; Stettler et al., 2016). Due to the similarity of the epitopes, ZIKV and DENV highly cross react with each other as observed in a handful of scientific studies (Priyamvada et al., 2016; Wen et al., 2017; Pardy and Richer, 2019). As DENV and ZIKV share genetic similarity and structural homology with each other, evidence indicate that these two viruses are also cross-reactive at the humoral level (**Figure 1**). The DENV infected patients have DENV antibodies that are partially cross-reactive against all four DENV serotypes and ZIKV as well. These antibodies tend to be low of avidity and weakly neutralizing. With the presence of ZIKV, many researchers have reported cross-reactivity between DENV and ZIKV sera (Wahala and Silva, 2011; Stettler et al., 2016).

The main epitopes identified to date include the EDII, EDIII, prM and the FLE (fusion loop epitope) (Wahala et al., 2009; Gerritsen and Pandit, 2015). What is also important to note is that a strong antibody response will neutralize the second virus and result in negligible disease, while a minimal antibody response may generate ADE conditions and this has been shown to occur in both these viruses. With regard to the innate immune responses using DENV-immune mice infected with ZIKV, the results showed no impact on the production of cytokines (IFN-y and TNF-α). However, unusual stimulations were detected by DENV- derived CD8+T cells which decreased ZIKV virus presence in the serum and brain samples of ZIKV infected mice (Wen et al., 2017). Conversely, a ZIKV-immune mice infected with DENV stimulated CD8+T cell responses against DENV using the peptides derived from prior ZIKV infection (Huang et al., 2017). Another study by Regla-Nava et al. (2018) (Regla-Nava et al., 2018) showed that pregnant DENV immune females were seen to be protected from fetal resorption caused by ZIKV infection by cross-reactive DENV specific memory CD8+T cells present in the body. Although, CD8+ cross reactivity is vastly observed in many studies, CD4+T cell cross reaction is not that common between ZIKV and DENV proteins. It has been shown that antigen-specific CD8+ T cells are made up largely of T_EM_ and T_EMRA_ subsets, but their relative distribution may vary considerably depending on the target antigen. T_EMRA_ cells are also considered by some authors to be the terminally differentiated effector cells supported by low Interleukin-2 and high interferon gamma secretion, high cytotoxicity, low proliferative capacity and high sensitivity to apoptosis (Graham et al., 2020). In a study to test the efficacy of a DENV vaccine, Graham et al. (2020) (Cimini et al., 2017) demonstrated that the protective DENV vaccine elicits multi-functional CD4+ and CD8+ T_EMRA_ cells and suggest that these virus-specific T cells may play a role in protective immunity. In past, Cimini et al. (2017) (Wahala and Silva, 2011) analyzed the similarity of CD8+ T cells and showed that there were no difference on the differentiation profile between CD8+ T cells of ZIKV and DENV. Thus, the actions of memory T cells against secondary infection and the cross reaction of CD4+T cell requires further explanation and studies to be understood fully.

## Future aspects

T cell subsets and biomarkers along with CTL CD4 cells discussed in this review, all show their own role in disease prevention in host immunity. However, their mechanisms are yet to be understood completely in the context of DENV and ZIKV infection in order to further use it in the disease treatment and prevention. Moreover, the disease similarity between DENV and ZIKV further indicates widely conserved epitopes and cross-reactive responses in the immune system and opens up for novel solutions in the world of vaccine design. Thus, further experiments and analysis are necessary. However, similarities or differences of CTL CD4 cellular responses between the 2 viruses is also important to be understood because both CD8+ and CD4+ immune cells collectively hold the potential to eliminate DENV and ZIKV infected cells and protect from the severity of the disease as stated by many researchers. Therefore, the mechanism of CTL CD4 response is essential to utterly understand the process of DENV and ZIKV elimination in order to produce effective and reliable vaccines against these multiple co-circulating infections caused by related Flavivirus.

## Author contributions

All authors listed have made a substantial, direct and intellectual contribution to the work, and approved it for publication.

## Funding

This work was supported by the Faculty of Medicine of University of Malaya, UM Frontier Research Grant FG016-17AFR.

## Conflict of interest

The authors declare that the research was conducted in the absence of any commercial or financial relationships that could be construed as a potential conflict of interest.

## Publisher’s note

All claims expressed in this article are solely those of the authors and do not necessarily represent those of their affiliated organizations, or those of the publisher, the editors and the reviewers. Any product that may be evaluated in this article, or claim that may be made by its manufacturer, is not guaranteed or endorsed by the publisher.
